# 125 Financial and prosocial incentives promote bike choices in a consequential laboratory task

**DOI:** 10.1093/eurpub/ckae114.067

**Published:** 2024-09-26

**Authors:** Valentina Kroker, Florian Lange

**Affiliations:** Ku Leuven, Leuven, Belgium; Ku Leuven, Leuven, Belgium

## Abstract

**Purpose:**

Incentives are a popular tool for fostering pro-environmental behavior. However, it is still largely unknown which types of incentives are most effective and whether their effectiveness persists beyond incentive discontinuation. To address this gap in literature, we designed an experiment, where we tested the comparative effectiveness of two different incentive structures in encouraging active and sustainable mobility in the laboratory. We expected that both financial and prosocial incentives would increase the number of bike choices in a consequential laboratory task compared to a no-incentive control group. To our knowledge, this has not been done in the pro-environmental behavior and active mobility literature.

**Methods:**

In a preregistered experiment, N = 238 participants were repeatedly given the choice to save actual energy at a real waiting-time cost in the Pro-Environmental Behavior Task. This was done by giving them the choice between the car option, which included shorter waiting periods but led to actual more CO2 emission, and the bike option, which always had a longer waiting time but did not emit extra CO2. We performed planned contrasts to estimate the effectiveness of the incentives compared to the control group.

**Results:**

Incentivizing participants with a 5-cent bonus payment (financial incentive) led to a significant increase in bike choices compared to a control group (d = 0.68). Similarly, incentivizing the energy-saving option with a 5-cent donation to charity (prosocial incentive) had a significant effect (d = 0.28). It therefore led to more bike choices in this incentive group compared to the control group. The difference between the incentive conditions was also significant (d = 0.41). In the second session, where we discontinued the incentives, the effect disappeared.

**Conclusion:**

These data illustrate the (temporally) effectiveness of financial and prosocial incentives. We show that incentives can encourage bike choices over car choices when incentives are employed. As a next step, we aim to test this in the field. A possible collaboration within the “Active Cities” project is being discussed at this moment.

**Support/Funding Source:**

This research was supported by a FWO postdoctoral fellowship (No 12U1221N) awarded to Florian Lange.

